# Observations on the Ossicula Auditus

**Published:** 1801-04

**Authors:** 


### *To the Editors of the Medical and Phyfical Journal.*

GENTLEMEN,

HEREWITH I tranfmit you a few Remarks on the Os Orbiculare Auris. They contain no new information relative to that bone; but as the publication of them may be the means of eliciting it from thofe who are better informed than myfelf, I fhall be highly obliged by the early infertion of them in your Journal. I am, your's, &c.

*Trowbridge, Mar.* 11, 1801. J. M. M.

---

The Officula Auditûs are ufually defcribed by modern Anatomifts as being four in number. With regard to two of thefe, the Incus and the Malleus, it is uncertain when, or by whom, they were difcovered. To Vefalius, they owe the names by which they are at prefent diftinguifhed. The Stapes appears to have been difcovered by Ingraffias, in the 15th century; though the difcovery has alfo been attributed to Euftachius, Columbus, and Vefalius. The Os Orbiculare was probably the laft of the four bones that was difcovered, Francis Sylvius being being the person to whom the discovery is attributed; though Morgagni thought that traces of a knowledge of it were to be found in the writings of Arantius.* Since the time of Sylvius, most Anatomists have considered the Os Orbiculare as a perfectly distinct bone, and one that is constantly to be met with in the human subject. I was surprised therefore to find, that Soemmerring, in his treatise *De Corporis Humani Fabrica*, notices among the Ossicula Auditûs only the Malleus, Incus, and Stapes. Of the last of these he says, "Minimum hocce totius corporis humani os." † And in describing the Incus he has these words, "e superficiei externæ (sc. *cruris anterioris*) extremo semiglobosum aut ovatum, numquam vel rarissime penitus separatum capitulum ope colli tenuis eminet,‡ quod noxum incudem inter et stapedem sistit."§ A few lines farther on he says, "Nonnumquam exiguum ossiculum malleum inter et incudem observatur." ||

The only modern author I have been able to consult, that agrees with Soemmerring is Jenty, who says, that the Os Orbiculare "is not truly a distinct or separate bone, but is merely an epiphysis of the longer leg of the Incus."** Jenty's work, however, is a mere compilation, and he has neglected, on this occasion, to give us his authority; so that it is impossible to determine what degree of weight must be attached to the remark. Few of your readers will be disposed to doubt the accuracy of Soemmerring; as few probably will be willing to allow his authority to outweigh that of almost all other Anatomists. In order, therefore, to determine whether the Os Orbiculare Auris ought to be reckoned among the bones constantly to be met with in the human subject, I beg leave to propose to practical Anatomists the following queries: 1st. Whether this bone, when it is met with, is not found to occupy different situations in different subjects? 2d. Whether it is not frequently wanting altogether?

If these queries should be answered in the affirmative, it will then appear that the Os Orbiculare is no more to be reckoned a distinct bone of the body than the Ossa Triquetra.

---

* Vid. Sabatier Traité Comp'et d'Anatomie, tom. ii. p. 342.

† De Corp. Human. Fab. tom. i. p. 136.

‡ "Vel in infante recens nato hoc ossiculum cum incude coalitum invenitur." *Camper Verhandelingen te Haaerlem*, 1767, Vol. viii. part 2, p. 209.

§ De Corp. Human. Fab. tom. i. p. 135.

|| Idem.

** Anatomico-Physiological Lectures, vol. i. p. 77.

*Extract*

